# Evaluation of restless legs syndrome symptoms in patients with and without the diagnosis of type 2 diabetes mellitus

**DOI:** 10.1186/1758-5996-7-S1-A89

**Published:** 2015-11-11

**Authors:** Arthur Onofre Beltran Filho, Guilherme Andreas Jung, Leandro Vicente Zoehler, Josue Cortez, Aline Vieira Scarlatelli Lima

**Affiliations:** 1 Universidade do Sul de Santa Catarina, Tubarão, Brazil

## Background

Restless legs syndrome (RLS) is a neurological sleep disorder. Literature suggests association between RLS and diabetes, but few studies have been done evaluating the association between these conditions[1].

## Objective

Evaluate the prevalence of RLS symptoms in patients with and without the diagnosis of Type 2 Diabetes Mellitus (DM2).

## Materials and methods

Case control study. 112 individuals were studied, 28 with DM2 (cases) and 84 without DM2 (controls), in a medical clinic specialities at Tubarão, Brazil. They answered a questionnaire with socio demographic questions, information about diabetes and to the four minimum criteria, defined by The International Restless Legs Syndrome Study Group (IRLSSG), for RLS diagnosis[2]. Furthermore, they were subjected to the Epworth Sleepiness scale and the IRLSSG rating scale, both translated and validated to Portuguese[3,4].

## Results

Among the cases, 21.4% presented RLS, compared to 14.3% in controls (p=0.269). The group with DM2 showed higher prevalence of Excessive daytime Sleepiness (EDS) (21.4% vs. 13.1%; p=0.219), and had higher scores in the IRLSSG rating scale (23,3±11,3 vs. 15,7±6,5; p=0.086). Correlating severity of RLS with glycemia, we obtained a Pearson correlation of 0.698 (p=0.003).

## Conclusions

DM2 patients have a higher, but non-significant prevalence of RLS symptoms and EDS than the non-diabetics. It has been found a correlation between RLS severity and glycemia.
